# Illegitimate and Repeated Genomic Integration of Cell-Free Chromatin in the Aetiology of Somatic Mosaicism, Ageing, Chronic Diseases and Cancer

**DOI:** 10.3390/genes10060407

**Published:** 2019-05-28

**Authors:** Gorantla V. Raghuram, Shahid Chaudhary, Shweta Johari, Indraneel Mittra

**Affiliations:** Translational Research Laboratory, Advanced Centre for Treatment, Research and Education in Cancer, Tata Memorial Centre, Navi-Mumbai 410210, India; vgorantla@actrec.gov.in (G.V.R.); schaudhary@actrec.gov.in (S.C.); sjohari@actrec.gov.in (S.J.)

**Keywords:** cell death, apoptosis, circulating nucleic acids, DNA damage, chromosomes, chromosomal damage, chromosomal mosaicism, inflammation, NFκB

## Abstract

Emerging evidence suggests that an individual is a complex mosaic of genetically divergent cells. Post-zygotic genomes of the same individual can differ from one another in the form of single nucleotide variations, copy number variations, insertions, deletions, inversions, translocations, other structural and chromosomal variations and footprints of transposable elements. High-throughput sequencing has led to increasing detection of mosaicism in healthy individuals which is related to ageing, neuro-degenerative disorders, diabetes mellitus, cardiovascular diseases and cancer. These age-related disorders are also known to be associated with significant increase in DNA damage and inflammation. Herein, we discuss a newly described phenomenon wherein the genome is under constant assault by illegitimate integration of cell-free chromatin (cfCh) particles that are released from the billions of cells that die in the body every day. We propose that such repeated genomic integration of cfCh followed by dsDNA breaks and repair by non-homologous-end-joining as well as physical damage to chromosomes occurring throughout life may lead to somatic/chromosomal mosaicism which would increase with age. We also discuss the recent finding that genomic integration of cfCh and the accompanying DNA damage is associated with marked activation of inflammatory cytokines. Thus, the triple pathologies of somatic mosaicism, DNA/chromosomal damage and inflammation brought about by a common mechanism of genomic integration of cfCh may help to provide an unifying model for the understanding of aetiologies of the inter-related conditions of ageing, degenerative disorders and cancer.

## 1. Introduction

Traditionally, human genetic research has been concerned with variations that are transmitted through the germ line. Recent research is, however, increasingly focusing on post-zygotic non-heritable genetic mutations that accumulate in the human soma throughout life leading to somatic mosaicism that ceases to exist following the death of an individual [1,2,3,4,5]. Mosaicism has been traditionally detected by cytogenetic analysis [5] and microarray-based studies [6,7]. More recently massively parallel next generation sequencing is being increasingly used to study somatic mosaicism [8,9]. It should be mentioned, however, that the concept of mosaicism is not new; in T cells, for example, acquired somatic gene rearrangements form the basis of diversity of immunoglobulin and T cell receptor genes [10].

Most types of mutations can cause somatic mosaicism [1]. Of these, structural or copy number variations (CNVs) are likely to lead to major somatic/chromosomal changes [5,6,11]. Point mutations in the form of single nucleotide variations (SNVs) or small insertion and deletions (indels) that largely arise from DNA replication errors are common causes of mosaicism [12,13]. These mutations arise as a consequence of errors during the repair of DNA damage, replication or mitosis [13]. They can also arise from intrinsic mutagens such as reactive oxygen species (ROS) as well as extrinsic agents, such as radiation and chemicals [14]. LINE1 transposable elements have been shown to cause mosaicism in the adult human brain [15,16] while Alu element retro-transposition has been detected in the brain and myocardium [17]. Tandem repeats have been shown to be polymorphic among individuals leading to somatic variations [18,19]. DNA repair by non-homologous end joining (NHEJ) can lead to insertions or deletions during DNA ligation [20]. Exchange of DNA between non-homologous repeats can also lead to large insertions or deletions [20]. Faulty DNA replication may cause complex rearrangement through multiple mechanisms [21] and mitotic mis-segregation of whole chromosome leading to chromosomal instability and aneuploidy is frequently seen in cancer [22]. Mosaicism of chromosomes contributes to ageing [23,24] and other diseases such as autoimmune disorders [25], autism [26] and schizophrenia [27]. It has been found to be a genetic cause of prenatal death [28] and specimens of spontaneous abortion have often revealed mosaic chromosomes [29]. Alzheimer’s disease [30] is frequently associated with an altered karyotype, suggesting similarities in the bases of chronic diseases and cancer [31]. Mosaic loss of chromosome Y detected in blood is associated with increasing age [32] and risk of cancer [33,34].

Single cell genetic analysis is revealing high levels of post zygotic variations in the brain, highlighting that no two cells in an adult brain may be genetically identical [35]. Sub-megabase CNVs have been shown to arise during cerebral neurogenesis in mice [36] and several studies have reported the presence of extensive post-zygotic CNVs and SNVs in neurons of the human brain [37,38,39]. A recent study of SNVs analysis by single-cell sequencing of 36 neurons of three normal individuals detected thousands of mutations [38]. These mutations appearing in non-dividing neuronal cells apparently reflect DNA damage during active transcription [38]. Another study reported that many neuronal cells contained at least one mega base size CNV, which may be related to neuro-psychiatric disorders [37]. A recent report has suggested a novel mechanism for generation of somatic mosaicism in the brain of healthy individuals and those with sporadic Alzheimer’s disease (SAD), which involves the reverse transcription and genomic re-integration of the Alzheimer’s disease-related (APP) gene [40]. Thousands of these integrated sequences occurring mosaically appeared as variant genomic cDNAs (gencDNAs). Neurons of patients with SAD showed increased gencDNA diversity as well as multiple mutations that are known to be associated with familial Alzheimer’s disease [40].

In blood cells, aberrant clonal expansion (ACEs) containing various mosaic genetic changes has been observed in normal individuals which increase with age [41,42] and appear to be related to haematological malignancies and cardiovascular disease [43]. In one study [41], structural variations in ACEs were observed in 10% of a population older than age 65 but in only 1% of those younger than age 50. A survey of 13 genome wide association studies detected ACEs in blood or buccal samples more frequently in cancer patients compared to healthy individuals in the form of aneuploidy and loss of heterozygosity [3]. Other studies have also reported an association between detectable ACEs in blood and risk of cancer [41,44]. Histologically normal cells in close vicinity to tumours show ACEs, suggesting that genetically aberrant cells precede cancer development [45]. Next generation sequencing analysis of somatic mutations in benign tissue adjacent to tumors found that 80% of samples contained clonal mutations which increased with age and smoking habits [46]. ACEs in blood have been shown to be associated also with non-cancer related diseases such as cardiovascular and Alzheimer’s disease and type 2 diabetes [43,47,48].

Genomic mosaicism can occur in both dividing and non-dividing cells [1]. Mosaicism in embryonic stem cells [49], as well as those in blood [41,42], occurs in proliferating cells leading to the formation of ACEs. On the other hand, much of the work on mosaicism has been done on neuronal cells which do not divide [15,16,37,38,39]. In addition to neuronal and hematopoietic cells, mosaicism has been observed in other tissues such as skin and oesophagus [50,51]. In a surprising finding, somatic mutations involving cancer related genes were uncovered in sun-exposed eyelid epidermis of normal individuals [50]. Deep sequencing of 74 cancer genes across 234 biopsies uncovered somatic mutations averaging two to six mutations per megabase per cell, which was similar to that seen in many cancers [50]. The extent to which cells in normal tissues accumulate mutations throughout life was further uncovered by genome sequencing of samples from the oesophagus of healthy individuals aged 20–75 years [51]. Somatic mutations were found to accumulate with age and a positive selection of clones carrying mutations in 14 cancer genes, with tens to hundreds of clones per square centimeter was discovered [51]. Unexpectedly, the prevalence of NOTCH1 mutations was several times higher in a normal oesophagus than in oesophageal cancers [51]. Taken together the above findings suggest that somatic mosaicism is prevalent in cells of all tissues of the body.

## 2. Cell-Free Chromatin (cfCh): Background

**Figure 1:**
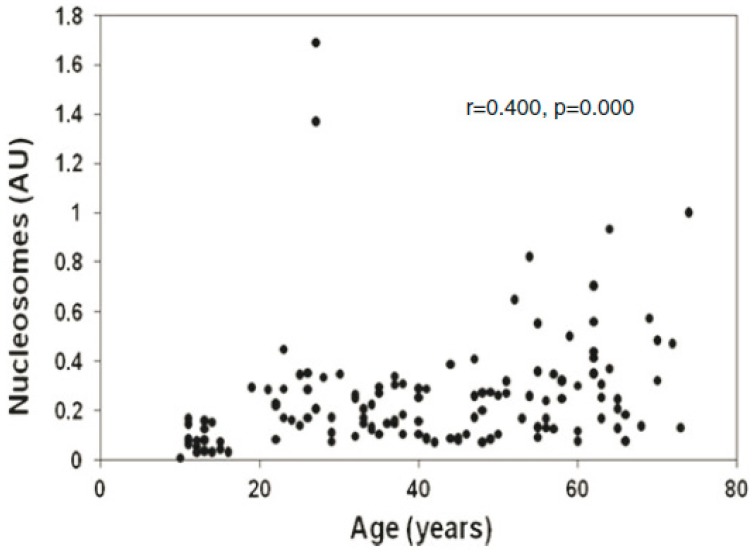
Levels of cell-free chromatin (cfCh) in serum increase with age. The study included 140 healthy subjects aged 15–70 years. The cfCh levels were measured using the Cell Death Detection ELISAPlus kit (Roche Apllied Sciences, Mannheim, Germany). Values are expressed as arbitrary units (AU). Reproduced from [62].

It has been estimated that 10^9^–10^12^ cells, primarily of haematogenous origin, die in the human body daily due to normal physiology [52], largely via apoptosis [53]. For example, the daily cellular turnover of granulocytes is 120 × 10^9^, of erythrocytes is 200 × 10^9^, of platelets is 150 × 10^9^ and of lymphocytes is 20 × 10^9^ [52]. Apoptotic cell death is characterized by nuclear and chromatin condensation and nuclear fragmentation [54]. Activation of endogenous nucleases, especially caspase-3 activated DNase, causes inter-nucleosomal cleavage of DNA leading to the formation of oligo-nucleosomes with multiples of 180–200 base pairs [55,56]. A significant number of nucleosomal fragments (cell-free chromatin, cfCh) thus generated enter into the extra cellular compartments of the body including into the circulation [57,58,59]. Levels of cfChs in blood are elevated in a multitude of human disorders such as cancer, inflammation and sepsis, cerebral stroke, trauma and auto-immune disorders ([60] for review). Increasing cfCh levels are positively associated with age [61,62] (Figure 1).

**Figure 2:**
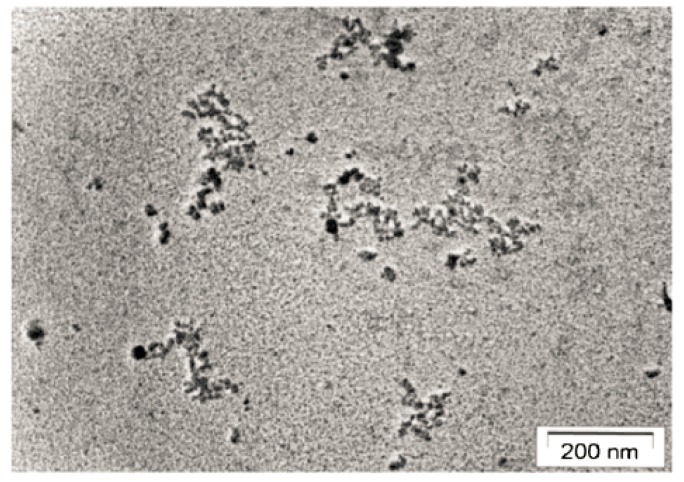
Electron microscopy image of cfCh isolated from pooled sera of cancer patients showing beads-on-a-string appearance typical of chromatin of disparate sizes. Reproduced from [64].

cfChs had a size range of 1–5 multiples of 185 bp–200 bp [63] and were successfully isolated from serum [64]. Electron microscopic examination of isolated cfCh from serum of cancer patients revealed a beads-on-a-string appearance, in which the beads were heterogeneous in size and ranged from ~10 nm > 1000 nm (Figure 2) [64]. There are several mechanisms by which the body attempts to eliminate cfCh. For example, dying cells are engulfed by phagocytes [65], while DNAse I present in blood attempts to inactivate cfCh by degrading its DNA component [66]. A decreased activity of DNase has also been observed in plasma from cancer patients, which might explain the elevated levels of cfCh found in cancer [67]. The half-life of cfCh has been estimated to be 10–15 min and it is continuously removed by the liver [68,69].

### 2.1. Cell-Free Chromatin Versus Cell-Free DNA

Although there is much current interest in cell-free DNA (cfDNA) as a biomarker in cancer diagnostics and therapy response [70], it is far from clear whether naked DNA circulates in the blood as a natural molecule. Cell death leads to inter-nucleosomal cleavage with release of mono- and oligo-nucleosomes [57,71] and not naked DNA. cfDNA that are detected in plasma or serum are likely to be a function of the DNA extraction process, which involves treatment with Proteinase-K [72]. On the other hand, the existence of cfCh in circulation can be directly detected by a sandwich ELISA assay which does not involve any extraction step [73]. It is likely that cfDNA in plasma/serum is generated from cfCh during DNA extraction since a strong positive correlation exists between circulating levels of cfCh and cfDNA [72]. Thus, while cfDNA may be useful a biomarker in cancer diagnostics, its biological relevance is questionable.

### 2.2. Uptake by Healthy Cells of cfCh Released into Circulation

In contrast to the many reports on the uptake of isolated DNA by cells ([74,75] for review), reports on the cellular uptake of cfCh are scant. Wagstaff et al. reported that in vitro reconstitution of DNA with histones produced chromatinized genes which were efficiently taken up by cells leading to their genomic integration [76]. The authors proposed that chromatinization of genes may be an efficient method for gene therapy. Although the existence of circulating cfCh has been known since the 1990s [77], whether cfCh has any pathophysiological role in the host has only recently been addressed [62,64]. Isolation of cfCh from sera of patients suffering from cancer and those from normal volunteers was reported for the first time in these studies [64]. The presence of cfCh in the isolates was confirmed by electron microscopy (Figure 2), Western blotting and agarose gel electrophoresis [64]. When the isolated cfCh were fluorescently dually labeled in their DNA and histones and added to NIH3T3 mouse fibroblasts cells in culture, numerous dually labeled fluorescent signals were seen in the nuclei of the recipient cells within minutes with a maximum uptake seen at six hours [64]. cfCh particles rapidly associated themselves with host cell chromosomes and activated a DNA damage repair (DDR) response which facilitated their incorporation into the host cell genomes by a unique mechanism (discussed later). Multiple proteins of the DDR pathway were up-regulated to include γH2AX, ATM, ATR, MDC-1, P-p53, P-p21, GADD-34, NIBRIN, RAD-50, MRE-11, DNA-PKcs and DNA ligase IV. Also, up-regulated were proteins of apoptotic pathways namely JC-1, cytochrome-C and caspase 3 [64].

**Figure 3:**
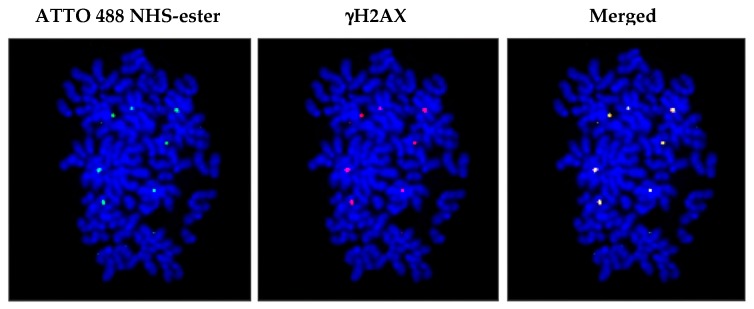
Genomic integration of cfCh in NIH3T3 cells leads to DNA double-strand breaks. NIH3T3 cells were treated with cfCh isolated from sera of cancer patients, labelled in their histones with ATTO 488 NHS-ester (ATTO-TEC GmbH, Germany. Catalogue No. AD 488-35) and applied to NIH3T3 cells. After several passages, metaphase spreads were prepared and immune-stained with antibody to γH2AX. Co-localization of green (cfCh) and red (γH2AX) signals are clearly seen under florescent microscopy. Magnification x60. Reproduced from [64].

The authors provided several lines of evidence to support their claim that cfCh had truthfully integrated into genome, and that the observed fluorescent signals were true reflections of the genomic integration of cfCh and not those of CfDNA [64]. First, several single cell clones were developed from NIH3T3 mouse fibroblast cells that were treated with cfCh and cfDNA isolated from sera of cancer patients and subjected to next generation sequencing. Tens of thousands of human reads were detected only in the cfCh derived clones, with a few human reads being detectable in the cfDNA clones [64]. The authors argued that the few integrated human reads in the cfDNA clones that were detected were a result of chromatinization of intracellular cfDNA particles with newly synthesized histones of the host cells [64]. These findings indicated that cfCh, rather than cfDNA, have the ability to efficiently integrate into host cell genomes. PCR amplification also detected sequences of multiple human Alu families in the cfCh clones [64]. Since, these single cell clones had been developed several years earlier and had undergone thousands of cell doublings, it is unlikely that the intracellular cfCh would remain extra-chromosomal without getting integrated into the host cell genomes. Second, NIH3T3 mouse fibroblast cells were treated with cfCh isolated from sera of cancer patients that had been dually labelled in their DNA with Platinum Bright™ 550 (red) and in their histones with ATTO 488 NHS-ester (green). After several passages, metaphase spreads were prepared from these cells and examined under fluorescent microscope. The red and the green signals invariably co-localized, indicating that the integrated particles were cfCh and not cfDNA [64]. Third, cfCh isolated from sera were labelled in their histones only with ATTO 488 NHS-ester and applied to NIH3T3 cells. Metaphase spreads were prepared and stained with antibody against γH2AX. Fluorescent microscopy revealed that the green signals representing cfCh co-localized with red signals of γH2AX, indicating that the act of genomic integration of cfCh had led to dsDNA breaks [64] and Figure 3.

**Figure 4:**
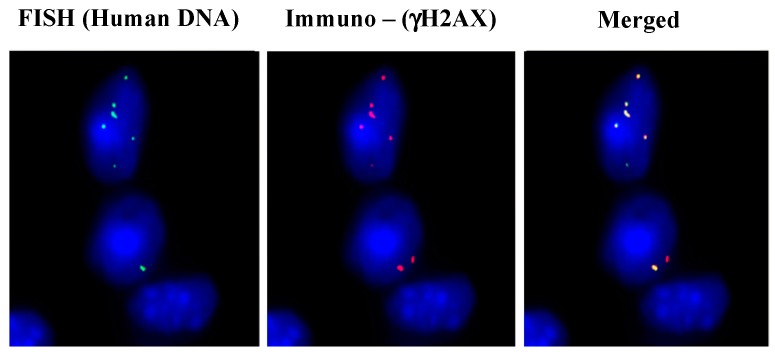
Genomic integration of cfCh in brain cells involves DNA double-strand break repair. Mice were injected intravenously with cfCh (100 ng DNA) isolated from cancer patients and sacrificed 24 h later. Sections of brain were processed for immuno-FISH using a human-specific whole genomic probe (green) and antibody against γ-H2AX (red). Co-localization of green and red signals are clearly visible. Magnification x60. Reproduced from [64].

Intravenous injection of cfCh isolated from sera of cancer patients in mice led to their integration into the nuclei of vital organs and they were detectable by FISH using human specific whole genomic and pan-centromeric probes [64]. Immune-FISH analysis revealed that the fluorescent human FISH signals in mouse brain cells co-localized with those of γH2AX, reconfirming their above in vitro finding that the act of genomic integration of cfCh leads to dsDNA breaks (Figure 4). The vital organs also showed extensive evidence of DNA damage and apoptosis when stained with antibody against γH2AX and active Caspase 3 [64].

Significantly, cfCh from cancer patients were found to be significantly more active than those isolated from healthy volunteers both in vitro and in vivo suggesting their possible role in cancer [64]. Thus, circulating cfCh may represent a new class of intra-corporeal mobile genetic elements [78] that act as continuously arising DNA mutagens [79]. Finally, all the above biological activities of cfCh could be abrogated by concomitant treatment with anti-histone antibody-complexed nanoparticles (CNPs) and DNase I both in vitro and in vivo [64,80].

### 2.3. Uptake by Healthy Cells of cfCh Released Locally from Dying Cells

**Figure 5:**
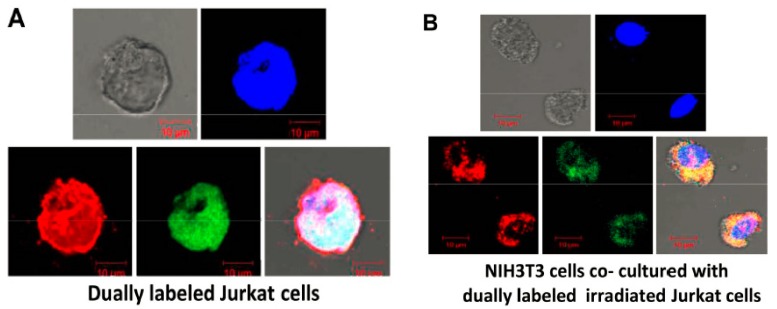
Confocal microscopy images to demonstrate bystander uptake by NIH3T3 cells of dually labeled cfCh particles released from irradiated dying Jurkat cells that had been labeled in their DNA with BrdU (red) and histones by CellLight^®^ Histone 2B-GFP (green). (**A**). dually labeled Jurkat cells. (**B**). NIH3T3 cells that had been co-cultivated with irradiated (15 Gy) dually labeled Jurkat cells at 24 h. Reproduced from [84].

Dying cells by apoptosis are normally phagocytosed by professional and non-professional phagocytes [81]. However, this mechanism is far from fool-proof [82]. Two recent studies have reported that cfCh released locally from dying cells can be ingested by bystander healthy cells [83,84]. Human lymphoblastic leukemia (Jurkat) cells were dually labeled in their DNA with BrdU and in their histone H2B with CellLight^®^ Histone 2B GFP (BacMam 2.0 - Thermo Fisher Scientific, Catalog number: C10594) and treated with ionizing radiation (15 Gy). When these dually labeled cells were co-cultured with NIH3T3 mouse fibroblasts, numerous dually labeled fluorescent particles were detected in the bystander NIH3T3 cells when examined by confocal microscopy at 24 h (Figure 5) [83,84]. The uptake of cfCh was dramatically reduced in the presence of cfCh inactivating agents namely CNPs, DNase I and a novel DNA degrading agent Resveratrol-copper (R-Cu) [83,85]. Thus, like circulating cfCh, those emerging from dying cells can spontaneously enter into healthy bystander cells [83,84].

Multiple human DNA signals were detected on metaphase spreads prepared from the co-cultured mouse fibroblast cells when examined by FISH [84], as was the presence of multiple human Alu sequences, confirming that *cfCh* from the dying human cancer cells had stably integrated into genomes of bystander mouse cells [83,84]. Genomic integration resulted in extensive chromosomal aberrations and instability [84]. It was also demonstrated that bystander uptake of cfCh can occur in distant organs [84]. Anaesthesized mice were delivered focused micro-beam irradiation (20 Gy) to the umbilical region and brain tissues were examined at 72 h. Intense activation of H2AX, active caspase 3, NFκB and IL-6 was observed [84]. All the radiation induced bystander parameters could be virtually abolished when the animals were concurrently treated with the three above mentioned cfCh inactivating agents [84].

### 2.4. Uptake of cfCh Released from Circulating Tumour Cells at Target Sites

**Figure 6:**
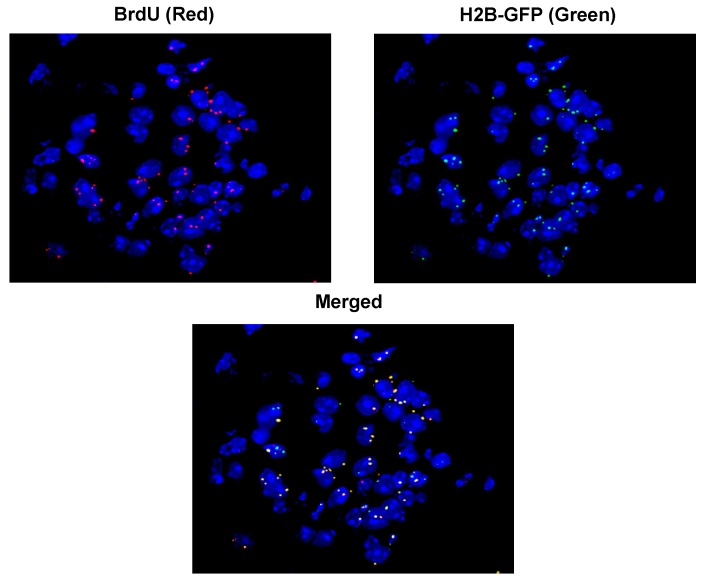
Detection of numerous fluorescent cfCh signals in nuclei of brain cells of mice following intravenous injection of fluorescently dually labelled MDA-MB-231 human breast cancer cells. MDA-MB-231 cells were dually labelled in their DNA with BrdU and in their histone H2B with CellLight^®^ Histone 2B GFP as described in [84]. One hundred thousand cells were injected intravenously, and animals were sacrificed after 72 h; sections of brain were examined by fluorescent microscopy as described in reference [64]. Magnification x60. (Unpublished data from authors’ lab).

Animal experiments have established that tumour cells undergo extensive cell death upon reaching target organs when injected intravenously into mice [86,87]. When MDA-MB-231 human breast cancer cells that had been dually fluorescently pre-labelled in their DNA and histone H2B were injected intravenously into mice, multiple dually labelled fluorescent signals were seen in brain cells (Figure 6). The cfCh fluorescent signals are seen to be strictly restricted within the nuclei of brain cells stained with DAPI, indicating that the injected cancer cells had undergone extensive cell death to release cfCh particles that had integrated into genomes of brain cells (Figure 6). This finding is consistent with earlier demonstration that cfCh has the ability to integrate host cell genomes [64].

**Figure 7:**
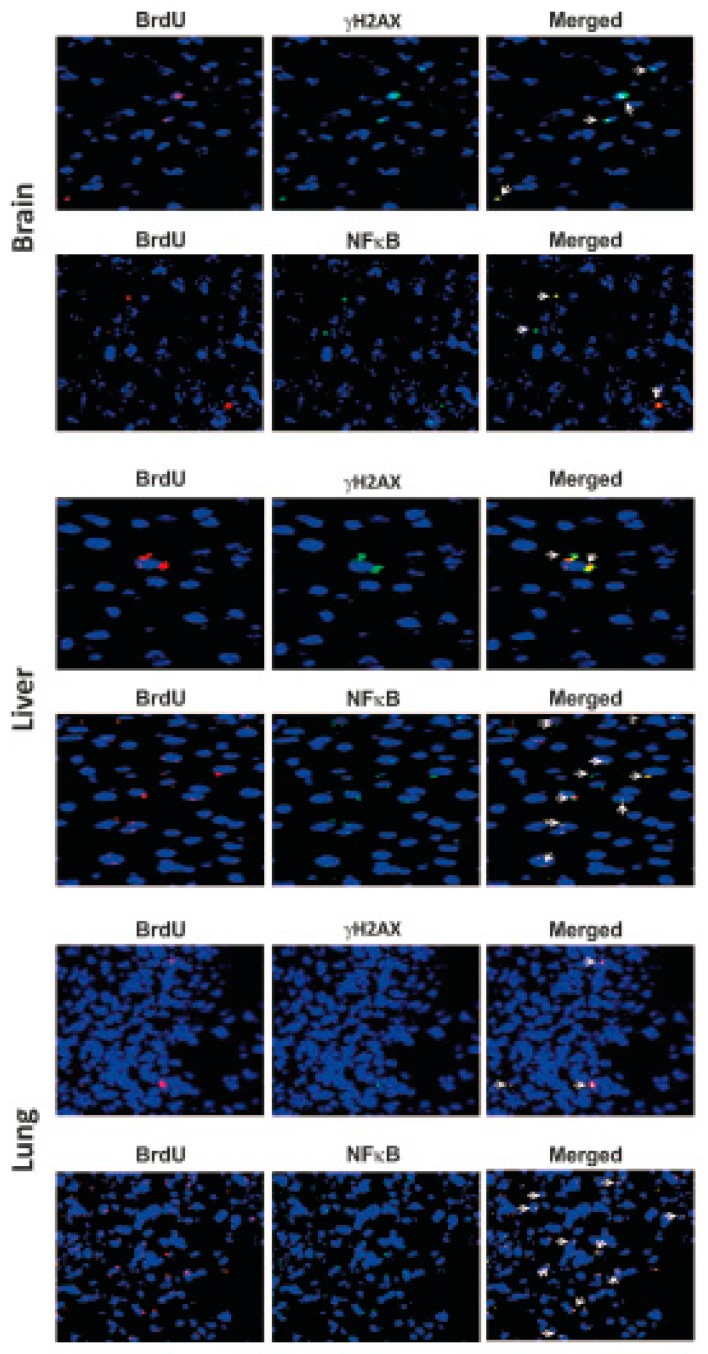
Co-localization of BrdU labelled fluorescent cfCh signals with those of γH2AX and NFκB in nuclei of cells of vital organs of mice. BrdU pre-labelled B16-F10 mouse melanoma cells were treated with Adriamycin and 10 × 10^4^ dying cells were injected intravenously. Animals were sacrificed after 72 h, vital organs were immuno-stained with antibodies against γH2AX and NFκB and examined by fluorescence microcopy. Magnification x40. Reproduced from [83].

The BrdU fluorescent signals representing cfCh derived from dying cancer cells co-localized precisely with those of γH2AX indicating that the act of genomic integration of cfCh particles had activated dsDNA breaks in cells of vital organs (Figure 7, upper panels of each image) [83]. Significantly the BrdU signals also co-localized with those of NFkB indicating the activation of inflammation (Figure 7, lower panels of each image) (discussed later). Concurrent treatment of mice with the cfCh inactivating agents viz CNPs, DNase I and R-Cu led to dramatic reduction in the number of γH2AX signals [83].

### 2.5. Mechanism of Genomic Integration of cfCh

**Figure 8:**
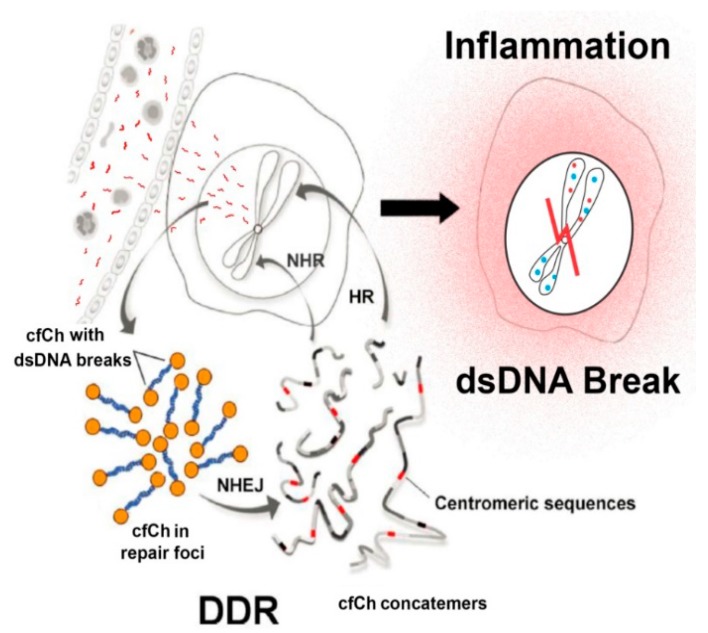
Schematic representation of a proposed model of DNA damage and inflammation following cellular uptake of cfCh. NHEJ = non-homologous end-joining; HR = homologous recombination; NHR = non-homologous recombination. Reproduced with modification from Chaudhary et al. 2018.

The authors of the above studies proposed a provocative model by which cfCh integrates illegitimately into genomes of local or distant bystander cells [64] (Figure 8). In this model DDR plays a crucial role and precedes DNA damage. In the classical model, DDR is activated after the occurrence of DNA damage in response to damaging agents such as ionizing and UV radiation and chemicals, free radicals etc. According to the new model this sequence is reversed; the acquired intracellular cfCh misleads the cell into perceiving them as fragments of its own chromosomes with broken DNA ends at each end. This leads the cell to mount a DDR/repair response well before any DNA damage having actually occurred. The DDR/repair response, which includes activation of DNA-PKc, DNA ligase IV and other repair proteins, links up the disparate intracellular cfCh fragments into long concatamers of discontinuous DNA segments which form new substrates for integration into host cell genomes, predominantly by NHEJ (Figure 8). Genomic integration of cfCh leads to dsDNA breaks and also of inflammation (discussed later).

**Figure 9:**
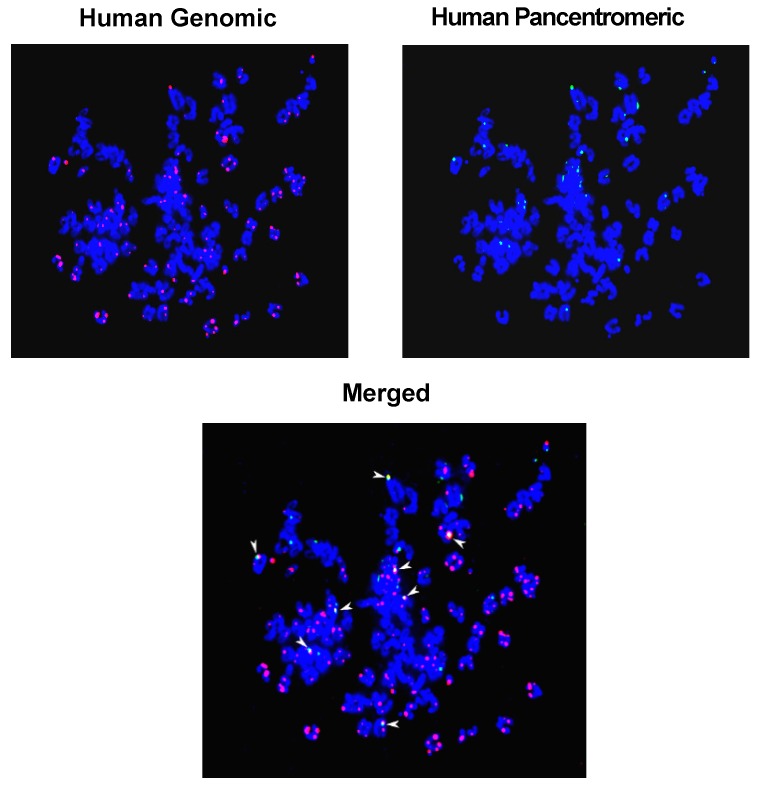
FISH detection of genomic integration of human DNA in mouse cells highlighting co-localization of human genomic and human pan-centromeric signals (arrow heads). Metaphase spreads were prepared from a single cell clone developed from NIH3T3 mouse fibroblast cells treated with cfCh isolated from sera of cancer patients [64]. FISH was performed using human whole genomic and human pan-centromeric probes as described in reference [64]. Magnification x60. (Unpublished data form authors’ lab).

The suggested formation of concatamers is supported by two observations: (1) given that the threshold value for detection of FISH signals is of the order of 30–50 kb [88], the detection of human DNA signals by FISH in mouse cells indicates that large human DNA segments, rather than discrete cfCh fragments, had integrated into mouse cell chromosomes; (2) combined FISH using human whole genomic and human pan-centromeric probes showed that genomic and centromeric signals frequently co-localize on chromosomal arms supporting the suggestion that the process of concatamerization of cfCh fragments can incorporate centromeric sequences within them (Figure 9) [64].

## 3. cfCh Integration, dsDNA Breaks and Somatic Mosaicism

**Figure 10:**
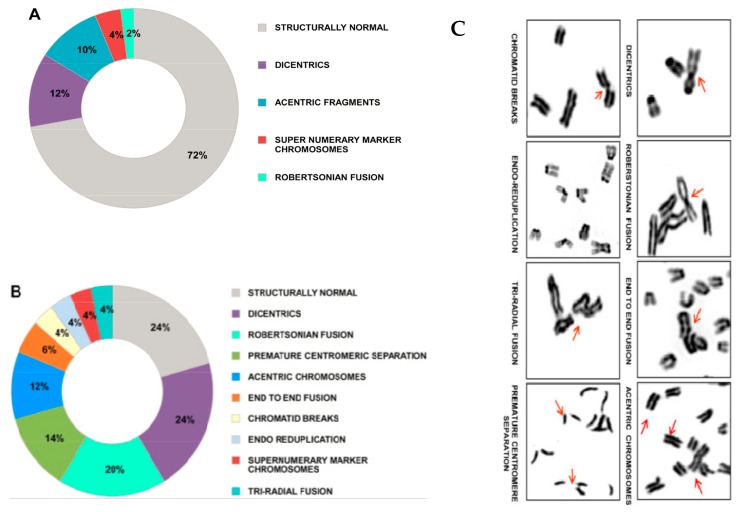
Chromosomal aberrations induced in healthy cells by cfCh released from dying cancer cells. NIH3T3 mouse fibroblast cells were grown in conditioned medium of irradiated (10 Gy) MDA-MB-321 human breast cancer cells for 96 h. Cell culture was continued in fresh medium and karyotype analysis was performed at the 10th passage. (**A**,**B**) wagon-wheel representation of chromosomal aberrations in control and conditioned medium treated NIH3T3 cells, respectively. Fifteen metaphases were analyzed in each case and average percentage values are given in the figures. (**C**) depiction of various chromosomal abnormalities. Reproduced from [84].

The model proposed above suggests a novel mechanism for the development of somatic mosaicism. Repeated and illegitimate genomic integration of cfCh and the resultant dsDNA breaks and their repair by NHEJ may generate a plethora of genomic variations that would increase with age. Age related mosaicism may be accelerated by the fact that cfCh levels in blood increases with age (Figure 1). Genomic integration of kilobase or megabase size concatamers will have major disruptive structural effects. Additionally, the concatamers themselves would comprise of a mosaic of discontinuous DNA segments adding to the complexity of mosaicism which would be further compounded by the presence of centromeric sequences within them. Furthermore, the fact that centromeric signals were detectable on chromosomal arms by FISH suggested that centromeric sequences themselves had undergone amplification/concatamerization during genomic integration to become visually detectable, thereby providing yet another facet to somatic mosaicism. The integrated concatamers would be repaired by NHEJ which is known to be error prone and can itself lead to insertions or deletions during DNA ligation as well as other genomic alterations such as translocations and chromosomal rearrangements [20,89,90,91]. Large scale integration of cfCh concatamers can cause physical damage to chromosomes leading to chromosomal aberrations and heterogeneity. Figure 10 depicts cytogenetic analysis of mouse fibroblast cells that had been grown in culture medium containing cfCh derived from irradiated dying human cancer cells. Multiple forms of chromosomal aberrations are seen in the mouse fibroblast cells which could lead to extensive chromosomal mosaicism and heterogeneity.

Somatic mosaicism is often seen in non-dividing cells, such as neurons [15,16,37,38,39,40,91]. Although it is believed that these are acquired during embryonic development when cells are dividing [92], the results presented above indicate that cfCh can integrate into the genomes of non-dividing cells of the adult brain by crossing the blood-brain barrier (Figure 4, Figure 6 and Figure 7). Specifically, Figure 4 and Figure 7 show that nuclei of non-diving mouse brain cells avidly incorporate cfCh particles that lead to dsDNA breaks. These data suggest that proliferation after acquiring mutations is not essential for the development of mosaicism. Illegitimate and repeated genomic integration of cfCh throughout life causing dsDNA breaks and repair by NHEJ, in addition to physical damage to chromosomes, will give rise to somatic mosaicism and chromosomal heterogeneity without the need for cellular proliferation. DNA/chromosomal damage can be mutagenic processes in cell cycle-arrested cells adding to their genetic complexity [91]. In conclusion, mutations of all varieties that characterize somatic/chromosomal mosaicism, in both dividing and non-dividing cells, can be generated by the repeated integration of cfCh or cfCh concatamers occurring throughout life.

## 4. cfCh Integration, dsDNA Breaks and Inflammation

The activation of inflammatory cytokines following the genomic integration of cfCh [83,84] has been alluded to earlier in this article. Co-cultivation of irradiated dying human cancer cells with mouse fibroblasts resulted in the uptake of numerous cfCh particles by the bystander cells to activate not only H2AX but also multiple inflammatory cytokines, which included NFκB, IL-6, TNF-α and IFN-γ [83]. All four inflammatory cytokines were up-regulated simultaneously to reach a maximum at ~6 hr which coincided with the maximum activation of γH2AX suggesting an inter-relationship between DNA damage and inflammation [83]. Further suggestion of a close relationship came from microarray analysis of the co-cultured cells which detected up-regulation of multiple pathways related to inflammation concurrently with those associated with cell cycle and DNA damage [83]. Finally, cfCh inactivating agents namely CNPs, DNase I and R-Cu not only prevented the activation of H2AX but also those of NFκB, IL-6, TNF-α and IFN-γ [83].

**Figure 11:**
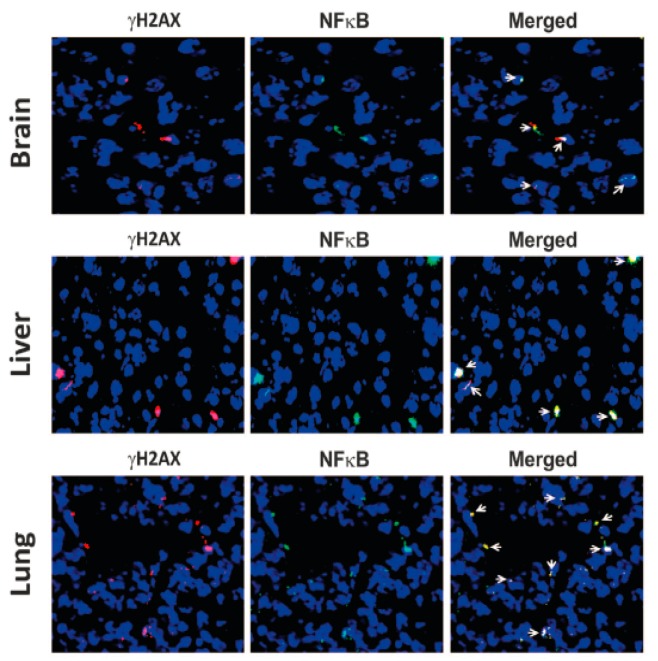
Co-localization of γH2AX and NFκB fluorescent signals in vital organs of mice following intravenous injection of Adriamycin treated dying B16-F10 cells. One hundred thousand cells were injected intravenously into mice and animals were killed after 72 h. The vital organs were removed and stained with antibodies against γH2AX and NFκB and examined by fluorescence microscopy. Magnification x40. Reproduced from [83].

As shown in Figure 7 earlier, intravenous injection of BrdU pre-labelled dying cancer cells led to uptake of cfCh by cells of vital organs followed by their genomic integration [83]. These experiments made the additional novel observation that the BrdU signals not only co-localized with those of γH2AX as mentioned earlier, but also co-localized with those of NFκB, indicating that the latter is activated at the sites of cfCh integration (Figure 7, lower panels of each image). More significantly, as shown in Figure 11, fluorescent signals of γH2AX and NFκB also co-localize strictly in the respective cellular nuclei, indicating that their activations are intimately inter-linked.

**Figure 12:**
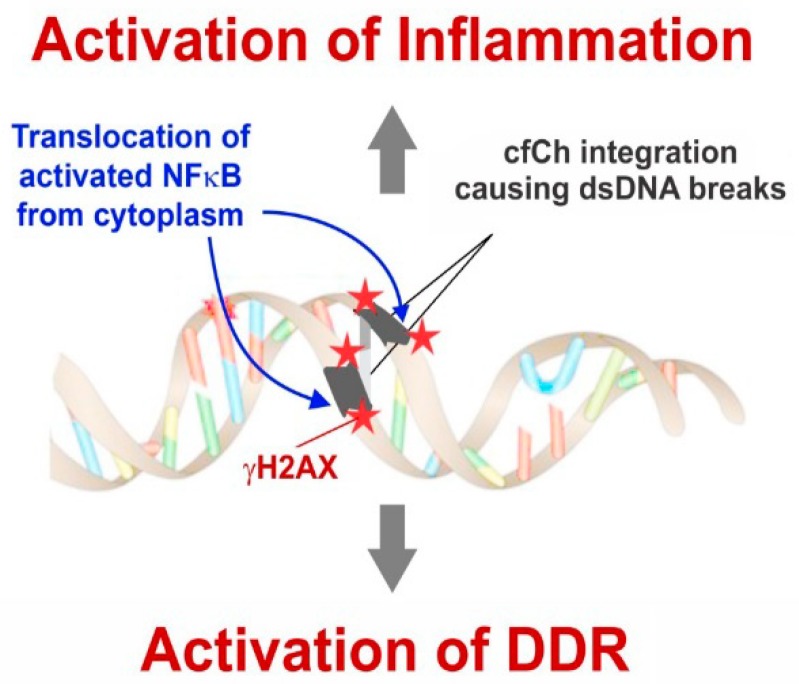
Schematic representation of a proposed model to explain detection of co-localized fluorescent signals of γH2AX and NFκB. Reproduced with modification from [96].

NFκB in an inactivated state remains sequestered in the cytoplasm [93]; however, it translocates to the nucleus upon activation by stressful stimuli such as DNA damage [94]. There have been several nuclear translocation sites reported for NFκB [95], but the finding, that γH2AX and NFκB fluorescence signals co-localize in nuclei of vital organs, leads us to propose that, following integration of cfCh and the consequent dsDNA breaks, NFκB is activated and then translocated from the cytoplasm to the specific cfCh integration sites in the genome [96] (Figure 12).

Other mechanism(s) of cfCh-induced inflammation need also to be considered. It is known that genomic stress, such as DNA damage, can lead to accumulation of DNA in the cytoplasm leading to activation of the DNA sensing GMP-AMP synthase stimulator of interferon genes (cGAS-STING)-mediated pathway to activate innate immune response and inflammation [97,98]. Several recent papers have implicated cytoplasmic chromatin fragments (CCF) in mediating immune activation [99,100,101]. cGAS-STING activation leads to two downstream pathways, namely, type I interferon through IRF3, and pro-inflammatory responses through NFκB [102]. Thus, in the current context, the possibility cannot be excluded that *cfCh* internalized into the cytoplasm may activate NFκB via the cGAS-STING pathway. However, and in any event, the observation depicted in Figure 11 and Figure 12, would suggest that NFκB thus activated via the cGAS-STING pathway would need to be translocated from the cytoplasm to the nucleus precisely to sites of *cfCh* integration.

**Figure 13:**
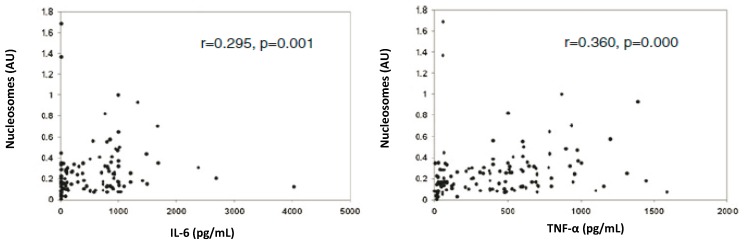
Positive correlation between serum cfCh levels and inflammatory cytokines (IL-6 and TNF-α). The study included 140 healthy subjects aged 15–70 years. cfCh levels were measured using the Cell Death Detection ELISAPlus kit (Roche Applied Sciences, Mannheim, Germany). Results are expressed in arbitrary units (AU). IL-6 and TNF-α were measured using cytometric bead array assay kit (BD Biosciences, San Jose, CA, USA). Reproduced from [62].

The mechanism of genomic integration of cfCh concatamers discussed earlier was depicted in Figure 8. The figure also incorporates the suggestion that genomic integration and the consequent dsDNA breaks may activate inflammation. Thus, cfCh arising from the billions of cells that die in the body everyday may act as continuously arising mediators of systemic inflammation by their ability to inflict dsDNA breaks in healthy cells of the body. It should be mentioned in this context that a strong positive correlation between blood levels of cfCh and inflammatory cytokines IL-6 and IFN-γ has been reported in healthy volunteers aged 50–75 years, reinforcing the suggestion of a close relationship between DNA damage and inflammation in human subjects (Figure 13) [62].

## 5. cfCh Induced Somatic Mosaicism, DNA Damage and Inflammation in Aetiology of Ageing, Chronic Diseases and Cancer

DNA sequence variations in the germ line are essential to promote evolution through natural selection. On the other hand, DNA sequence variations in post-zygotic tissues may have deleterious consequences for the host. The post-zygotic genome may accumulate mutations throughout life to an extent that no two genomes of an individual are identical with each other [1,2,3,4]. In spite of a vast literature speculating on the causes of somatic mutations, no unifying theme has emerged that would explain the plethora of genomic variations that are seen in somatic genomes. Accumulating mutations, and the accompanying somatic mosaicism, is considered to be a major underlying cause of ageing [42,43] and age-related disorders such as type 2 diabetes [47], cardiovascular disease [43] Alzheimer’s disease [48], and cancer [41,44,50,51].

The discovery that cfCh that emerge from the billions of the cells that die in the body every day can integrate into host cell genomes and inflict dsDNA breaks [64,83] has important implications for development of genomic mosaicism and disease processes. Genomic integration of cfCh (or their concatamers) into healthy cells and the consequent dsDNA breaks and repair by NHEJ may bring about the diverse genetic alterations that underlie somatic mosaicism. DNA and/or chromosomal damage triggered by cfCh integration may also activate an inflammatory response, characterized by the induction of multiple cytokines. Significantly, the master transcription factor NFκB translocates into the nucleus to co-localize at the sites of dsDNA breaks resulting from cfCh integration, suggesting that inflammation may be a direct response to dsDNA breaks [96]. Thus, while the integration of cfCh and the resulting DNA damage may bring about the diverse genomic changes that underlie somatic mosaicism, inflammation provides a new facet to these complex processes that may be key to disease pathologies. DNA damage and sterile inflammation are associated with cardiovascular [103,104] and Alzheimer disease [105,106], type 2 diabetes [107,108], cancer [109,110] and above all ageing [111,112]. Thus, the triple pathologies of somatic mosaicism, DNA/chromosomal damage and inflammation brought about by a common mechanism of genomic integration of cfCh may help to provide a unifying model for the understanding of aetiologies of the inter-related conditions of ageing, degenerative disorders and cancer.
